# The Prostaglandin E2 Pathway and Breast Cancer Stem Cells: Evidence of Increased Signaling and Potential Targeting

**DOI:** 10.3389/fonc.2021.791696

**Published:** 2022-01-19

**Authors:** Olivia L. Walker, Margaret L. Dahn, Melanie R. Power Coombs, Paola Marcato

**Affiliations:** ^1^ Pathology, Dalhousie University, Halifax, NS, Canada; ^2^ Biology, Acadia University, Wolfville, NS, Canada; ^3^ Microbiology and Immunology, Dalhousie University, Halifax, NS, Canada

**Keywords:** prostaglandin E2, breast cancer, cancer stem cells, aldehyde dehydrogenase, CD44+/CD24-, EP receptors

## Abstract

Culprits of cancer development, metastasis, and drug resistance, cancer stem cells (CSCs) are characterized by specific markers, active developmental signaling pathways, metabolic plasticity, increased motility, invasiveness, and epithelial-mesenchymal transition. In breast cancer, these cells are often more prominent in aggressive disease, are amplified in drug-resistant tumors, and contribute to recurrence. For breast cancer, two distinct CSC populations exist and are typically defined by CD44+/CD24- cell surface marker expression or increased aldehyde dehydrogenase (ALDH) activity. These CSC populations share many of the same properties but also exhibit signaling pathways that are more active in CD44+/CD24- or ALDH+ populations. Understanding these CSC populations and their shared or specific signaling pathways may lead to the development of novel therapeutic strategies that will improve breast cancer patient outcomes. Herein, we review the current evidence and assess published patient tumor datasets of sorted breast CSC populations for evidence of heightened prostaglandin E2 (PGE_2_) signaling and activity in these breast CSC populations. PGE_2_ is a biologically active lipid mediator and in cancer PGE_2_ promotes tumor progression and poor patient prognosis. Overall, the data suggests that PGE_2_ signaling is important in propagating breast CSCs by enhancing inherent tumor-initiating capacities. Development of anti-PGE_2_ signaling therapeutics may be beneficial in inhibiting tumor growth and limiting breast CSC populations.

## Introduction

Breast cancer is the most commonly diagnosed cancer among women, with nearly a quarter of all patients eventually succumbing to the illness (1–3). There is a need to increase understanding of this disease with the intent that it will lead to development of improved therapeutic strategies and outcomes. In terms of impact on cancer initiation, development, progression and drug resistance, cancer stem cells (CSCs) have an important role. Possessing stem-like characteristics with increased tumorigenicity, these cells have the capacity to self-renew and differentiate into bulk tumor cells (4–6).

CSC-associated enzymes and signaling pathways may provide novel avenues for therapeutic intervention, since these pathways and enzymes are also mediators of tumorigenicity, metastasis, and therapy resistance. Among the most well-studied breast CSC-associated signaling pathways are the developmental Notch, Wnt and Hedgehog pathways (7–9). There are clinical trials underway evaluating therapeutics which target elements of these pathways and they have been reviewed extensively elsewhere (7, 10–13). In this review, we consider the emerging evidence of the role of the prostaglandin synthesis pathway in breast CSC maintenance, assess published transcriptome data for evidence of increased prostaglandin synthesis pathway activation in breast CSC populations, and discuss how this pathway could be targeted in the treatment of breast cancer and limitation of CSC populations.

## The Prostaglandin Synthesis Pathway

Prostanoid prostaglandins and thromboxanes are a class of biologically active lipids that belong to the eicosanoid family (14). They are released in response to tissue trauma and are mediators of pain, inflammation, fever, and uterine contractions. The prostanoids are critical in the maintenance of gastric function and renal blood flow (14) and they initiate cell signaling events that result in changes to cell proliferation, apoptosis, differentiation, and adhesion (15–18).

**Figure 1:**
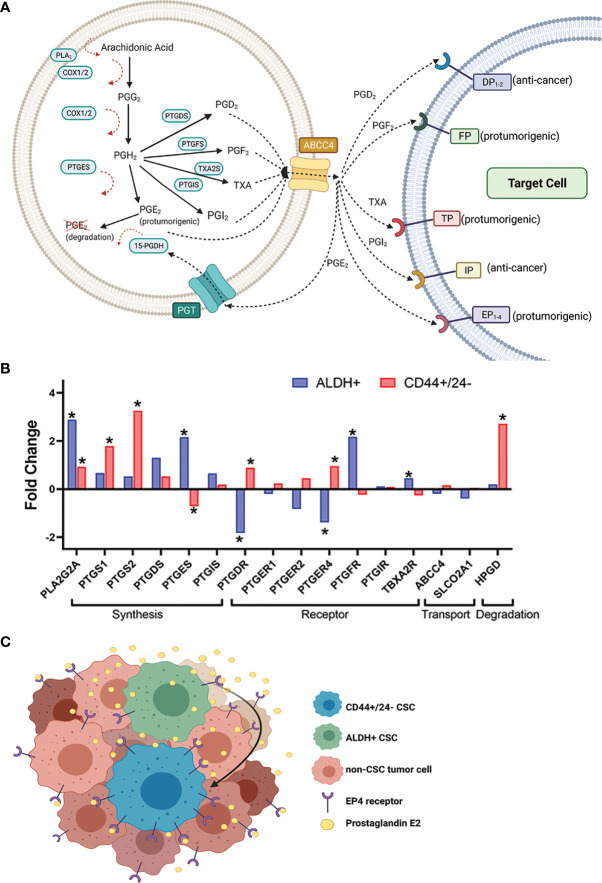
Expression of certain prostaglandin pathway genes in sorted CSC populations from breast cancer patient tumors suggests a hypothetical model for heightened CSC-PGE_2_ signaling in breast tumors. **(A)** Overview of the key players in the synthesis of the prostanoids and subsequent prostaglandin signaling **(B)** Fold change in expression of the prostaglandin synthesis and signaling genes in breast cancer patient tumor cells sorted for ALDH+ versus ALDH- (GSE52327); or CD44+/CD24- versus non-CD44+/CD24- (GSE5713) cells. Significance is noted by *, which represents a p-value <0.05. If more than probe was identified for a gene, the data for the probe with the most significant fold change is shown. Analyzed genes include phospholipase A (*PLA2G2A*), COX-1 (*PTGS1*), COX-2 (*PTGS2*), prostaglandin D synthase (*PTGDS*), prostaglandin E synthase (*PTGES*), prostacyclin synthase (*PTGIS*), DP receptor (*PTGDR*), EP receptors 1, 2,4 (*PTGER1, 2,4*), FP receptor (*PTGFR*), IP receptor (*PTGIR*), TP receptor (*TBXA2R*), MRP4 (*ABCC4*), PGT (*SLCO2A1*), 15-PGDH (*HPGD*). Data for the probes against *PTGFS* and *PTGER3* were missing and so they not included in the analysis. Probe IDs for each of the genes included in the analysis are 243928_s_at *ABCC4*; 203913_s_at *HPGD*; 203649_s_at *PLA2G2A*; 215894_at *PTGDR*; 212187_x_at *PTGDS*; 214391_x_at *PTGER1*; 206631_at *PTGER2*; 204897_at *PTGER4*; 207388_s_at *PTGES*; 1555097_a_at *PTGFR*; 206187_at *PTGIR*; 211892_s_at *PTGIS*; 205128_x_at *PTGS1*; 204748_at *PTGS2*; 204368_at *SLCO2A1*; 207555_s_at *TBXA2R.* **(C)** Hypothetical model based on the data from **(B)**, where increased PGE_2_ signaling in the breast tumor microenvironment results from the interplay between ALDH+ and CD44+/CD24- CSC populations.

The prostanoids are produced in multi-step processes involving multiple enzymes (**Figure 1A**). First, arachidonic acid is liberated from phospholipid bilayers by phospholipase A_2_ (PLA_2_) and is oxidized by prostaglandin-endoperoxide synthase 1 and 2 (PTGS1 and PTGS2), which are commonly referred to as the cyclooxygenases: COX-1 and COX-2. The COX enzymes generate precursor prostaglandin G2 (PGG_2_) and then prostaglandin H2 (PGH_2_) (19, 20), and are differentially expressed in different tissue types (14). COX-1 is constitutively expressed at low levels in most tissues and by maintaining prostanoid production at basal levels, it contributes to gastric function and renal blood flow homeostasis (14, 21, 22). In contrast, COX-2 is not constitutively expressed and is induced during inflammation and in response to mitogens (21–24).

After the rate-limiting steps of the COX enzymes, the multiple prostaglandin synthase isoforms PTGIS, TXA2S, PTGES, PTGDS, and PTGFS generate the different prostanoids: prostaglandin I2 (PGI_2_, also called prostacyclin), thromboxane A2 (TXA_2_), prostaglandin E2 (PGE_2_), prostaglandin D2 (PGD_2_), and prostaglandin F2 alpha (PGF_2α_), respectively from PGH_2_ (**Figure 1A**). The prostanoids are secreted by the multidrug resistance protein 4 (MRP4/ABCC4) and exert effects by binding to and activating specific G-protein coupled receptors expressed on target cells (**Figure 1A**) (25). Secreted PGE_2_ acts in paracrine or autocrine fashion by binding prostaglandin E2 receptors 1, 2, 3 and 4 (EP1-4), which are respectively encoded by the *PTGER1, PTGER2, PTGER3*, and *PTGER4* genes (**Figure 1A**) (26, 27). PGE_2_ signaling termination is mediated by the prostaglandin transporter (PGT) and 15-hydroxyprostaglandin dehydrogenase (15-PGDH) (28). PGT allows the uptake of extracellular PGE_2_, which is then degraded by 15-PGDH.

As summarized in **Table 1**, the prostanoids have both overlapping and at times unique and counteracting activities. PGE_2_ is by far the most implicated of the prostanoids in terms of importance in cancer and will be the major focus of the following sections.

**Table 1:** Summary of the function of the prostanoid products, their receptors, and role in cancer.

Prostanoid	Function	Receptors	Involvement in cancer	Sources
TXA_2_	Prothrombotic	TXA_2_ receptor (TP)	Promotes cancer cell proliferation, migration (breast), angiogenesis (endothelial, melanoma, lung)	(29–31)
	Platelet aggregation			
PGI_2_	TXA_2_ antagonist	PGI_2_ receptor (IP)	Anticancer, prevents metastatic tumor formation, decreases angiogenesis (breast and melanoma)	(31–33)
	Haemostasis			
	Antithrombotic			
	Vasodilator			
PGD_2_	Inhibits platelet aggregation	PGD_2_ receptor 1 and 2 (DP_1,2_)	Decreased angiogenesis (mast cell activation)	(34, 35)
	Anti-inflammatory		Decreased proliferation and metastasis by reducing expression of TWIST2 (breast)	
	Immunomodulatory, IgE mediated Type 1 allergy through mast cell activation			
PGF_2_	Cell proliferation by inducing MAPK signaling cascades	F prostanoid receptor (PGF_2α_/ FP)	Increased cell proliferation through EGFR and MAPK pathways (breast and uterus)	(36–39)
	Ovulation, uterine contractions, luteolysis			
	Arterial contractions			
	Pain sensing			
PGE_2_	Pain sensing Uterine contractions	PGE_2_ receptors 1-4 (EP1-4)	Promotes cancer cell proliferation, migration, invasion (bladder, breast, kidney)	(15, 20, 23, 24, 40, 41)
	Renal haemostasis		Increased mesenchymal phenotype (breast)	

The cancer studied is indicated in parentheses.

## Prostaglandin E2 in Cancer

As the primary mediator of the oncogenic effects of COX-2, PGE_2_-induced receptor signaling contributes to almost all of the major cancer hallmarks, including angiogenesis, proliferation, epithelial–mesenchymal transition (EMT), and the maintenance of CSC characteristics (42, 43). PGE_2_ is the most common prostanoid in the tumor microenvironment (44, 45). In breast cancer there is a well-established paradigm of elevated COX-2, low 15-PGDH, increased PGE_2_, and associations with more aggressive disease and negative outcomes (43, 44, 46–49). COX-2 overexpression induces tumorigenesis *via* PGE_2_ production, increased angiogenesis (42), and suppressed tumor immunity (40, 43–45, 50, 51). In patient tumors, COX-2 overexpression is associated with poor prognoses and lower distant disease-free survival time (52). In contrast, the PGE_2_ degrading enzyme 15-PGDH is tumor suppressive, where its downregulation results in enhanced breast cancer cell proliferation, cell cycle entry *in vitro*, and enhanced tumorigenicity *in vivo* (28, 46, 53, 54). Non-transformed healthy cells have high cellular levels of 15-PGDH and corresponding low levels of PGE_2_ (19); comparatively, cells with low levels of 15-PGDH and corresponding high levels of PGE_2_ have enhanced tumorigenicity (55). The deregulation of genes like *PTGS2* (COX-2) and *HPGD* (15-PGDH) could be the drivers behind the prostaglandin-induced cell changes associated with cancer.

Among the PGE_2_ receptors, EP2 and EP4 have the most important roles in breast cancer (56, 57). PGE_2_ signaling through EP2 can increase tumor angiogenesis and proliferation (20, 26); and can modulate metabolism and promote tumorigenesis through EP4 (26, 58). Stimulation of EP2 increases vascular endothelial growth factor (VEGF) signaling in COX-2 transgenic murine mammary tumors, leading to increased angiogenesis and lymphangiogenesis (59). Additionally, EP2 silencing reduces proliferation and invasion by decreasing the expression of matrix metallopeptidases 2 and 9 (MMP2 and MMP9), and cyclin D; conversely, EP2 overexpression increases tumor volume and metastasis by activating MMPs (60). In *in vivo* models, metastatic sites had significantly increased levels of EP2 (60).

Binding of PGE_2_ to EP4 leads to dramatic changes in cell biology, especially in the context of cancer. PGE_2_ binding to EP4 stimulates the reorganization of actin stress fibres and focal adhesion complexes responsible for epithelial cell attachment to the basement membrane of the extracellular matrix (61). In breast cancer, EP4 stimulation increases proliferation and invasiveness (62), promotes lymphangiogenesis and metastasis (63), and is associated with aggressive phenotypes (64). Breast cancer cell treatment with an EP4 agonist or synthetic PGE_2_ both stimulated epidermal growth factor receptor (EGFR) and increased tumor spheroid invadopodia, invasion, and extracellular matrix degradation by breast cancer cells (65).

In terms of the downstream molecular signaling events that lead to these EP2 and EP4-mediated phenotypes, EP2 and EP4 both signal through a protein-kinase A (PKA)-dependent manner to increase intracellular levels of cAMP (26, 66); EP4 also signals through the phosphatidylinositol 3-kinase (PI3K)/Akt pathway (58, 66, 67). The stimulation of both receptors can have similar outcomes; however, EP4 stimulation seems to have a more important role in promoting stemness in cancer cells because it can signal through both cAMP/PKA and PI3k/Akt/NOTCH/WNT pathways in COX-2 positive cells. PGE_2_ stimulation of EP4 through both PKA and PI3k/Akt pathways inhibit glycogen synthase kinase-3 (GSK3), which acts as a negative regulator of Wnt and Notch pathways thereby activating these pathways and inducing the expression of genes associated with stemness, cell cycle, angiogenesis, and lymphangiogenesis (58, 63, 68). Furthermore, induction of hypoxia-inducible factor (HIF)-1 in tumors results in expression of genes involved in angiogenesis, glucose metabolism, and cell survival in breast cancer cells. The synthesis of HIF-1 is regulated by the PI3K and MAPK pathways (55) through which EP4 signals (26, 58). Therefore, PGE_2_ activates multiple pathways through EP4 that are involved in cancer cell migration and proliferation.

The PGE_2_ signaling pathway appears to have greater importance in the triple-negative breast cancer (TNBC) subset of breast cancers (64, 69). In addition to staging, breast cancers have distinct prognoses and treatment strategies based on the expression of the hormone receptors estrogen receptor (ER), progesterone receptor (PR), and human epidermal growth factor receptor 2 (HER2). This sub-classifies breast cancers as ER+/PR+, HER2+, or TNBC (those lacking expression of the three receptors). TNBCs represent 10-15% of breast cancers that do not respond to endocrine therapies, have poorer outcomes, and are in most need of novel targeted therapies (70). In a comprehensive study evaluating the players of the PGE_2_ pathway in breast cancer patient tumors, MRP4, PGT, and 15-PGDH were noted as differentially expressed among distinct breast cancer subtypes (64). High PGE_2_ in the TNBC tumor microenvironment could be promoted by a combination of high COX-2, high MRP4, low PGT, and low 15-PGDH which was observed in TNBCs (64, 71).

The breast patient tumor data is reflected in cell lines. Secreted PGE_2_ levels are high in cultured TNBC cells (69, 72). Key for the high secretion of PGE_2_ by TNBCs is the combination of high MRP4, low PGT, and low 15-PGDH typically found in cell lines of this subtype (64, 69). This is also reflected in breast cancer cell lines; TNBC MDA-MB-231 cell have high EP4, MRP4 and COX-2, while ER+ MCF7 cells have comparably low levels of these PGE_2_ pathway players (71). It is also noteworthy that TNBCs are enriched for both CD44+/CD24- and aldehyde dehydrogenase positive (ALDH+) breast CSCs compared to other molecular subtypes (73–80), hence the greater impact the PGE_2_ pathway has in TNBC may also be in part connected to effect on CSC populations.

### Evidence for the Role of Prostaglandin Signaling in Breast Cancer Stem Cells

In a 1980 study of breast tumors, Rolland et al. found that in breast cancer patients, high levels of prostaglandins were found in tumor cells present in the lymphatics and nodal tissues (81). They suggested that the tumor cells with high prostaglandin synthesis/response were involved in progressing disease by enhancing migration from the established primary tumor to secondary sites (81). They proposed the existence of a subpopulation of breast tumor cells with high prostaglandin levels that are responsible for driving disease progression; in hindsight they may have been referring to CSCs, which were discovered a couple decades later in leukemia (4, 6).

In 2003, the existence of a highly tumorigenic sub-population of breast cancer cells displaying stem-like characteristics and identified based on CD44+/CD24- cell surface expression was first reported (82). A few years later, a second method for identifying breast CSCs was proposed; increased ALDH activity detected by the Aldefluor assay (83), typically imparted by increased levels ALDH1A3 and/or ALDH1A1 isoforms (84). It is noteworthy that CD44+/CD24- or Aldefluor+/ALDH+ breast cell populations only partly overlap and in fact the two CSC populations have some distinct properties. CD44+/CD24- breast CSCs are more mesenchymal and are associated with tumorigenesis and proliferation; whereas ALDH+ CSCs are more epithelial and are associated with increased metastatic capacity (78, 85–87). The enhanced plasticity of CSCs permits the distinct CD44+/CD24- or ALDH+ CSC populations to transition between the two CSC states which best fits the conditions of the tumor microenvironment. COX-2 derived PGE_2_ may be a contributing factor to the development of breast CSCs that are associated with tumor initiation (82) and chemotherapy resistance (88, 89). As there exists two distinct populations of CSCs in breast cancer, when reviewing the literature for the potential role of prostaglandin signaling in breast CSC phenotypes it is important to define how the CSCs were identified since there may be a CD44+/CD24- or ALDH+ specific effect.

More studies evaluating PGs in breast CSCs have thus far focused on the CD44+/CD24- phenotype. For example, fibroblasts secreting high levels of PGE_2_ had enhanced tumor growth and increased proportion of CD44+/CD24- cells (90), and the ability to secrete PGE_2_ was associated with the ability to expand CD44+/CD24- breast cancer cells *in vivo* (90). EP4 inhibition of CD44+/CD24- high TNBC MDA-MB-231 cells depleted their drug efflux transporters and CSC-associated proteins like CD44, β-catenin, and fibronectin by inducing their secretion in extracellular vesicles (71). Treatment of ER+ MCF7 cells with the extracellular vesicles from MDA-MB-231 cells transferred the mesenchymal CSC/mesenchymal attributes to MCF7 cells (71). Taken together these studies indicate that increased PGE_2_/EP4 signaling promote the tumor and mesenchymal activities of CD44+/CD24- breast CSCs; however, any corresponding effect on the more epithelial-like ALDH+ CSCs was not specifically assessed in the studies.

There is evidence of ALDH+ breast CSC associations with increased PGE_2_ signaling (56, 58). An *in situ* analysis of breast tumor tissue revealed increased EP4 and COX-2 expression in patients was correlated with increased ALDH1A expression and reduced patient survival (58). COX-2 overexpression in ER+ MCF7 and HER2+ SKBR3 cells increased their ALDH activity, the number of ALDH+ cells, and phenotypes characteristic of CSCs (EMT, spheroid formation, expression of stemness markers, tumorigenicity) (58). These changes were reversed upon treatment with COX-2 inhibitors or EP4 antagonists (58). They also found high expression of COX-2 and EP4 in ALDH+ breast cancer cells. Together the data suggest that ALDH+ CSCs produce more COX-2 derived PGE_2_ and upregulate the receptors that respond to PGE_2_ in a way that promotes cell growth and survival (58). EP4 may be a valuable target for ablation of ALDH+ breast CSCs.

The data discussed so far is mostly generated from analysis of breast cancer cell lines; analysis of sorted breast CSCs from patient tumors in the context of prostaglandin synthesis and signaling in breast CSCs would provide additional valuable information. Furthermore, there is no direct comparison yet published assessing how the manipulation of the prostaglandin synthesis pathway may affect CD44+/CD24- versus ALDH+ breast CSC populations. In advance of such evidence, we note that that there are published breast cancer patient tumor transcriptome data available which provides an opportunity for hypothesis generation. We accessed GSE7513 and GSE52327, which consist of gene array data from breast cancer patient tumor cells sorted for CD44+/CD24- or ALDH+ from cells lacking these marker profiles. We specifically assessed the expression of the prostaglandin synthesis and signaling genes in these datasets to test if elements of this pathway are of greater importance in one CSC population versus the other (**Figure 1B**). We filtered the dataset to focus specifically on the genes in the prostaglandin synthesis pathway (**Figure 1B**).

This analysis revealed that some of the genes in the prostaglandin pathway are differentially expressed in CSC populations versus non-CSC populations from patient tumors. In both the ALDH+ and CD44+/CD24- sorted cells, *PLA2G2A* (encodes PLA_2_) is upregulated (**Figure 1B**). Given that PLA_2_ is involved the first step of the synthesis pathway, the upregulation of this gene provides the possibility for greater availability of precursor for potential prostaglandin generation.

More striking is the distinct prostaglandin pathway genes that are differentially expressed in the ALDH+ population compared to CD44+/CD24- sorted patient breast cancer tumor cells. Of note, the genes that encode the rate-limiting COX enzymes *PTGS1* and *PTGS2*, are significantly upregulated in CD44+/CD24- populations. The generator of key pro-tumorigenic PGE_2_, *PTGES* is significantly upregulated in ALDH+ cells and conversely downregulated in CD44+/CD24- sorted tumor cells. In contrast, *PTGER4* (encodes EP4, the critical receptor for the pro-tumorigenic autocrine and paracrine PGE_2_ signaling) is downregulated in ALDH+ cells and conversely upregulated in the CD44+/CD24- cells (**Figure 1B**). Finally, *HPGD* which encodes the PGE_2_ signal terminator 15-PGDH is significantly upregulated in the CD44+/CD24- tumor cells; this could negate any increased production of PGE_2_ cells due to increased PLA_2_/COX-2, since increased 15-PGDH would degrade produced PGE_2_. In contrast, there is not significant fold change difference in *HPGD* levels in ALDH+ over ALDH- cells (**Figure 1B**).

Together this patient tumor data of CSC sorted populations leads us to hypothesize that if breast CSCs are playing an important role in (and promoted by) heightened PGE_2_ signaling, there may be a complex interplay in the tumor microenvironment between these two CSC populations (**Figure 1C**). Potentially ALDH+ CSCs are producing/secreting more PGE_2_ and the CD44+/CD24- CSCs may be benefiting from this *via* increased expression of receptor EP4 and paracrine signaling. We hypothesize that in a heterogeneous tumor, certain populations of CSCs may be secreting inflammatory, pro-tumorigenic factors into the tumor microenvironment which can be utilized by all cells in the tumor environment, including other CSC populations to support tumor growth and invasiveness (**Figure 1C**).

### Prostaglandins in Breast CSCs: A Druggable Target?

Taken together, the published studies in the role of prostaglandins in breast cancer and CSCs suggest that targeting this prostaglandin pathway could limit CSC numbers while inhibiting the cancer overall. Treatment with a COX-2 inhibitor can sensitize chemoresistant breast cancer cells to chemotherapy drugs like paclitaxel or doxorubicin (91). This is consistent with prostaglandins playing a key role in chemoresistance and suggests that disrupting the synthesis or signaling of pro-tumorigenic prostaglandins could be a valuable treatment option for reducing drug resistance and CSC development. However, there is a lack of published research investigating the role of PGE_2_ in breast CSC-mediated drug resistance. One study reported that celecoxib treatment decreased CSC markers and reduced EMT gene signatures in TNBC cell lines (92). This could be important in the context of treatment as the lethality and progression of most patients’ cancers may be reduced through the prevention of an invasive mesenchymal phenotype.

Eicosanoid products are key signaling molecules and modulators of inflammation and pain; they have been the target for pain management drugs like non-steroidal anti-inflammatory drugs (NSAIDs). Inhibition of COX enzymes by NSAIDs are commonly used to control pain and inflammation, but they also prevent the downstream formation of all prostanoid products which can be harmful to the patient. The inhibition of COX-2 activity with long-term use of NSAIDs can have an ulcerative effect on intestinal epithelia (22), is associated with renal toxicity (93), and puts patients at risk of developing cardiotoxicity like blood clots, hypertension, or stroke (19). Therefore, it would be beneficial to find a target further downstream in the prostaglandin synthesis pathway that allows for the blockade of pro-tumorigenic PGE_2_ without disrupting the cardioprotective prostanoids.

Enzymes involved in PGE_2_ synthesis and signaling or EP4 receptors have been suggested as drug targets (28) but none are currently approved for clinical use. Inhibition of EP4 by pharmacological agents inhibits the growth of mammospheres, as determined by decreased cellularity *in vitro*, and *in vivo* treatment with EP4 antagonists reduced the tumor-forming capacity of breast cancer cells with reduced frequency of CSCs in the tumor and lung metastases (94). EP4 antagonists were as effective as celecoxib at reducing the rate of tumor growth, stem cell markers, spontaneous lung and lymph node metastases, angiogenesis, and lymphangiogenesis (56). This suggests that downstream targets for PGE_2_ could be as effective as COX-2 inhibitors without the potential side effects.

## Targeting the Prostaglandin Synthesis and PGE_2_ Pathway in Breast Cancer: Clinical Trial Data

There have been several clinical trials using the COX-2 inhibitor celecoxib completed in the treatment of breast cancer (95, 96). One study showed promising results with decreased metastasis, angiogenesis, and inflammation when given perioperatively (96). In a phase II trial, pre-treatment and post-treatment breast biopsies revealed that celecoxib induces transcription of genes associated with anti-tumour and decreased cell proliferation (97). However, in another study patients treated long-term with celecoxib had no significant difference in disease-free survival compared to placebo group (95). Hence, further studies are needed to clarify the potential clinical impact of targeting this pathway. Additionally, inhibiting COX-2 lacks the specificity of targeting the PGE_2_ pathway specifically, thereby possibly limiting potential therapeutic efficacy with increased potential for the adverse side-effects as described above. It is noteworthy that in these celecoxib clinical trials, the COX-2 inhibitor was well-tolerated, with low rates of reported toxic effects.

Looking ahead, it appears that the clinical data targeting the PGE_2_ pathway may be forthcoming as several early phase clinical trials are underway investigating EP4 antagonists in cancer. Specific to breast cancer, is a new Phase II trial investigating EP4 antagonist grapiprant in the treatment of metastatic inflammatory breast cancer (NCT05041101). The trial is recruiting patients with a proposed completion date of December 31, 2025. An increase in overall and progression-free survival in patients that receive grapiprant would provide impetus that further clinical investigation is warranted.

## Challenges, Advantages, and Limitations

The pre-clinical data supports investigating targeting PGE_2_ in the treatment of breast cancer, with effects on CSCs. Realistically, obtaining the clinical data that demonstrate therapeutic efficacy of this approach has challenges to overcome and is many years away. Like most clinical trials, we are limited by the potential for demonstrated efficacy based on the eligibility of the patient population enrolled in the trial. For example, having advanced disease which has been refractive to treatment with standard of care is typically an eligibility requisite and this may also limit evidence of therapeutic efficacy. Additionally, a lack of consideration of the tumor composition of the enrolled patients may limit efficacy. Consideration for levels of PGE_2_, COX-2, EP4, and CSCs present in pre-treatment tumors when enrolling patients may increase the number of patients that achieve objective responses.

Most of the completed pre-clinical breast cancer studies investigating the inhibition of PGE_2_ downstream of COX-2 have focused on EP4 signaling, yet PGE_2_ signals through four EP receptors, and while the data thus far suggests that EP4 is the most important in terms of cancer, signaling through EP2 activates some stemness pathways. By focusing solely on EP4, the full potential of specific PGE_2_ inhibition may be missed.

Specific to our understanding of the role of the prostaglandin signaling pathway in breast CSCs, we are limited by the lack of data utilizing heterogeneous patient tumors and studies that compare the effects of the prostaglandin pathway in both the CD44+/CD24- ALDH+ breast CSC populations in the same study. Given that these CSC populations have distinct properties, this will be necessary to gain a full understanding of the impact of the pathway in the context of breast CSC signaling.

## Conclusions

Overall, the evidence strongly suggests that the prostaglandin pathway (specifically PGE_2_ signaling) in breast cancer is tumor-promoting and enhances the CSC phenotype. The effects of enhanced PGE_2_ signaling appears to be of greater magnitude in CSC-rich TNBCs. A lack of published research directly comparing the ALDH+ and CD44+/CD24- breast CSC populations in the context of patient tumor samples indicates an opportunity for further study to elucidate the mechanism of prostaglandin signaling in breast CSCs and the tumor microenvironment. Other than one study of fixed patient breast tumors (58), most work on the role of prostaglandins in breast CSCs has been conducted using cell lines. Experiments investigating the role of anti-PGE_2_ treatment using patient-derived xenografts, followed by a limiting dilution assay to assess CSC numbers would clarify the potential importance of the prostanoid in breast tumors and CSCs.

Other treatment targets, such as specific PGE_2_ receptor antagonists or synthesis inhibitors (for example, against PTGES), may be valuable in the prevention of tumor proliferation, invasion, and metastasis of breast cancer. PGE_2_ appears to be important in the progression of many cancer types, so, studying the effect of anti-PGE_2_ treatment in breast cancer may be advantageous to inform potential broad-spectrum anti-cancer options. The prostaglandin pathway (beyond PGE_2_-EP4 signaling specifically) could be an important focus in the treatment of breast cancer, especially for TNBCs or tumors enriched in CSCs.

## Author Contributions

OW and PM drafted the manuscript and conceptualized the key points. MD and MP provided expert feedback and edited the manuscript. All authors contributed to the article and approved the submitted version.

## Funding

OW is supported by a Genomics in Medicine scholarship from the Dalhousie Medical Research Foundation (DMRF) and a Nova Scotia Graduate Scholarship. Costs of publishing this review are covered by a Canadian Institutes of Health Research grant (CIHR, PJT 162313) to PM.

## Conflict of Interest

The authors declare that the research was conducted in the absence of any commercial or financial relationships that could be construed as a potential conflict of interest.

## Publisher’s Note

All claims expressed in this article are solely those of the authors and do not necessarily represent those of their affiliated organizations, or those of the publisher, the editors and the reviewers. Any product that may be evaluated in this article, or claim that may be made by its manufacturer, is not guaranteed or endorsed by the publisher.
